# Semisupervised Kernel Marginal Fisher Analysis for Face Recognition

**DOI:** 10.1155/2013/981840

**Published:** 2013-09-12

**Authors:** Ziqiang Wang, Xia Sun, Lijun Sun, Yuchun Huang

**Affiliations:** School of Information Science and Engineering, Henan University of Technology, Zhengzhou 450001, China

## Abstract

Dimensionality reduction is a key problem in face recognition due to the high-dimensionality of face image. To effectively cope with this problem, a novel dimensionality reduction algorithm called semisupervised kernel marginal Fisher analysis (SKMFA) for face recognition is proposed in this paper. SKMFA can make use of both labelled and unlabeled samples to learn the projection matrix for nonlinear dimensionality reduction. Meanwhile, it can successfully avoid the singularity problem by not calculating the matrix inverse. In addition, in order to make the nonlinear structure captured by the data-dependent kernel consistent with the intrinsic manifold structure, a manifold adaptive nonparameter kernel is incorporated into the learning process of SKMFA. Experimental results on three face image databases demonstrate the effectiveness of our proposed algorithm.

## 1. Introduction

During the past decade, face recognition has been an active area of research in image processing and computer vision due to its extensive range of prospective applications, such as human-computer interface, information surveillance, and identity authentication. One of the most successful and well-studied techniques to face recognition is the appearance-based method. When using appearance-based methods, a face image of size *n*_1_ × *n*_2_ pixels is usually represented by a vector in an *n*_1_ × *n*_2_-dimensional space. Consequently, the face images are typically of very high dimensionality, ranging from several thousands to several hundreds of thousands. Due to the consideration of the curse of dimensionality, learning in such high dimensionality in many cases is computationally expensive and often leads to low recognition accuracy. One common response to address this problem is to apply dimensionality reduction techniques to generate a lower-dimensional equivalence of the original high-dimensional face image space for the given observations and targets. Once the high-dimensional face image data is projected into lower-dimensional feature subspace in which the semantic structure of the face image space becomes clear, traditional classification schemes can then be applied. To this end, principal component analysis (PCA) and linear discriminant analysis (LDA) [1] are the most well-known dimensionality reduction techniques.

PCA aims to find a set of mutually orthogonal basis vectors that capture the global information of the data points in terms of variance, and the orthogonal basis vectors are the leading eigenvectors of the data's total variance matrix associated with the leading eigenvalues. PCA is optimal in terms of representation and reconstruction, but not for discriminating one face class from others. Unlike PCA, which is unsupervised, LDA is a supervised dimensionality reduction algorithm. LDA aims to find an optimal transformation that maps the data into a lower-dimensional space that minimizes the within-class scatter and simultaneously maximizes the between-class scatter, thus achieving maximum discrimination. Both PCA and LDA have widely been applied to face recognition and image retrieval. It is generally believed that, when they come to solving problems of pattern classification, LDA-based algorithms outperform PCA-based algorithms since the former focuses on the most discriminant feature extraction while the latter achieves simply object reconstruction [2]. Independent component analysis (ICA) [3] is another linear subspace analysis method, which separates the high-order moments of the input data besides the second-order moments in PCA. However, previous researches reported that ICA gave the same recognition accuracy as PCA, sometimes even a little worse than PCA [4]. In addition, NMF is also a subspace method which aims to find a parts-based representation of objects by imposing nonnegative constraints [5]. However, NMF is an unsupervised learning method and still focuses on the global geometrical structure of face image space. Moreover, the iterative update method for solving NMF problem is computationally expensive. In summary, the aforementioned algorithms see only the global Euclidean structure and can not discover the local manifold structure hidden in the high-dimensional data. In fact, a number of research efforts have shown that the face images possibly reside on a nonlinear submanifold hidden in the face image space [6–13]. Therefore, face representation is fundamentally related to the problem of manifold learning.

Manifold learning focuses on uncovering the compact, low-dimensional representations of the observed high-dimensional data that lie on or nearly on a manifold in an unsupervised manner. In order to detect the underlying manifold structure, many manifold learning algorithms have been proposed, such as isometric feature mapping (ISOMAP) [14], local linear embedding (LLE) [15], and Laplacian eigenmap (LE) [16]. ISOMAP, a variant of multidimensional scaling (MDS), aims to perverse global geodesic distances of all pairs of samples. LLE is based on the assumption that data lying on a nonlinear manifold can be viewed as linear in local areas, and it aims to discover the nonlinear structure via locally linear reconstructions. LE aims to preserve proximity relationships by manipulations on an undirected weighted graph, which indicates neighbour relations of pairwise data points. Thus, one of the key ideas of these manifold learning algorithms is the so-called locally invariant idea [17]; that is, the nearby points are likely to have the similar embedding/labels. Despite that these manifold learning algorithms have yielded impressive results on some benchmark artificial data set, they suffer from the out of sample problem; that is, they yield maps that are defined only on the training data points and how to evaluate the maps on novel test data points remains unclear. Therefore, these manifold learning algorithms might not be optimal in discriminating face images with different semantics, which is the ultimate goal of face recognition. To cope with the out of sample problem, He and Niyogi [18] applied a linearization procedure to construct explicit maps over new samples and proposed locality preserving projection (LPP) algorithm for manifold learning. LPP is a linearization of LE, aims to discover the local geometrical structure, and can be derived by finding the optimal linear approximations to the eigenfunctions of the Laplace-Beltrami operator on the manifold. As LPP is unsupervised, it is designed to best preserve data locality or similarity in the embedding space rather than good discriminating capability. As a result, the projected data points of different classes may still mix up after LPP embedding, which deteriorates the discrimination performance. In other words, for classification problem such as face recognition, the local manifold structure itself is not sufficient. A successful manifold learning algorithm should have the following two properties: (1) close intraclass pairs remain close after projection and (2) close but dissimilar pairs are kept separate after projection. Based on this consideration, Yan et al. [19] recently proposed the marginal Fisher analysis (MFA) method for manifold learning by simultaneously utilizing the local manifold structure and the class label information. The empirical studies in [19] have shown that MFA is more competitive than LDA and LPP algorithms on face recognition.

MFA is a supervised learning method. It searches for the projection directions on which the marginal sample pairs of different classes are far away from each other while requiring data points of the same class to be close to each other. To obtain good generalization capability on testing samples, one needs a collection of labelled data points to train MFA. However, in the many practical applications of pattern classification (such as face recognition), one often faces a lack of sufficient labelled data, since labelling often requires expensive human labour and much time. Meanwhile, large numbers of unlabeled data can be far easier to obtain. Given the high cost in manually labelling face image data and at the same time abundant unlabeled face image is often easily accessible, it is desirable to develop dimensionality reduction methods that are capable of exploiting both labelled and unlabeled data. This motivates us to introduce semisupervised learning [20] into the dimensionality reduction process.

All the early semisupervised learning techniques mainly focus on semisupervised classifier design [21–25], which aims to employ a large number of unlabeled data to help build a better classifier from the labelled data. Recently, the semisupervised learning idea has been successfully applied to feature selection [26], clustering [27], distance metric learning [28], and matrix factorization [29]. Particularly, the semisupervised learning idea achieved great successes on various image analysis tasks. For example, semisupervised discriminant analysis (SDA) [30] used the consistency assumption; that is, nearby samples in the feature space or samples on the same manifold structure are likely to have the similar embedding/labels. All these approaches demonstrated that the learning performance can be significantly enhanced if the consistency assumption is exploited and the unlabeled data is considered. It is very natural that this idea should also be considered in semisupervised dimensionality reduction. However, most of the existing extension algorithms of MFA fail to take into account the intrinsic manifold structure revealed by unlabeled data points.

In this paper, we propose a novel semisupervised kernel MFA (SKMFA) algorithm, which takes advantage of both labelled and unlabeled data for face recognition. The main idea of our algorithm is to convert the traditional marginal Fisher analysis (MFA) into a semisupervised kernel counterpart, which still has no straightforward solution available in the literature. In addition, for semisupervised kernel MFA, the kernel function has an essential impact on the dimensionality reduction performance. Therefore, we propose to first induce a new manifold adaptive kernel by employing kernel deformation techniques to incorporate the manifold structure revealed by unlabeled data into the nonparameter kernel and then apply semisupervised kernel MFA to dimensionality reduction tasks by using the manifold adaptive kernel. Finally, extensive experiments on three face image databases demonstrate the effectiveness of the proposed SKMFA algorithm.

The rest of the paper is organized as follows. In Section 2, we provide a brief review of marginal Fisher analysis (MFA) algorithm.  Section 3 introduces our proposed semisupervised kernel MFA (SKMFA) algorithm for face recognition. The experimental results on face recognition are presented in Section 4. Finally, we provide the concluding remarks and suggestions for future work in Section 5.

## 2. Brief Review of MFA

Marginal Fisher analysis (MFA) [19] is a recently proposed manifold learning algorithm for dimensionality reduction; it is based on the graph embedding framework and can precisely model both intraclass compactness and interclass separability by jointly considering the local manifold structure and the label information, as well as characterizing the separability of different classes with the margin criterion. Meanwhile, MFA avoids the out of sample problem existing in traditional manifold learning algorithms by applying a linearization procedure to construct explicit maps over new samples.

Given a set of face images {*x*_1_, *x*_2_,…, *x*_*n*_} ⊂ ℝ^*p*^, let *X* = [*x*_1_, *x*_2_,…, *x*_*n*_]; there are *c* classes; the *i*th face image *x*_*i*_ is associated with a class label *c*_*i*_ ∈ {1,2,…, *c*}. MFA aims to find a linear transformation *U* ∈ ℝ^*p*×*q*^ that maps each face image *x*_*i*_  (*i* = 1,…, *n*) in the *p*-dimensional space to a vector *y*_*i*_ in the lower *q*-dimensional space by *y*_*i*_ = *U* ^*T*^ *x*_*i*_ such that *y*_*i*_ represents *x*_*i*_ well in terms of maximizing the interclass separability and simultaneously minimizing the intraclass compactness. The optimal linear transformation of MFA can be obtained by solving the following maximization problem:

(1) Uopt =argmaxUS~pS~c =argmaxU∑i∑(i,j)∈Pk2(ci)  or  (i,j)∈Pk2(cj)||UTxi−UTxj||2Wijp    ∑i∑i∈Nk1(j)  or  j∈Nk1(i)||WTxi−WTxj||2Wij =argmaxUUTX(Dp−Wp)XTUUTX(D−W)XTU,

where ${\tilde{S}}_{p}$ and ${\tilde{S}}_{c}$ denote the interclass separability and intraclass compactness, respectively, and their definition, are as follows:

(2) S~p=∑i∑(i,j)∈Pk2(ci)  or  (i,j)∈Pk2(cj)||UTxi−UTxj||2Wijp=2UTX(Dp−Wp)XTUS~c=∑i∑i∈Nk1(j)  or  j∈Nk1(i)||WTxi−WTxj||2Wij=2UTX(D−W)XTU,

where *W*_*ij*_^*p*^ and *W*_*ij*_ denote the weighting coefficients of penalty graph and intrinsic graph defined on the data points, respectively; *W*_*ij*_^*p*^ and *W*_*ij*_ as well as their corresponding diagonal matrices *D* ^*p*^ and *D* are defined as follows:

(3) $$\begin{array}{c} W_{ij}^{p}=\{\begin{array}{cc} 1, & \text{if}\;\left(i,j\right)\in P_{k_{2}}\left(c_{i}\right)\;\;\text{or}\;\;\left(i,j\right)\in P_{k_{2}}\left(c_{j}\right)\\ 0, & \text{otherwise} \end{array}\\ W_{ij}=\{\begin{array}{cc} 1, & \text{if}\;i\in N_{k_{1}}\left(j\right)\;\;\text{or}\;\;j\in N_{k_{1}}\left(i\right)\\ 0, & \text{otherwise} \end{array}\\ D_{ii}^{p}=\sum_{j}W_{ij}^{p}\\ D_{ii}=\sum_{j}W_{ij}, \end{array}$$

where *P*_*k*_2__(*c*_*i*_) denotes a set of data pairs that are the *k*_2_ nearest pairs among the set {(*i*, *j*) | *c*_*i*_ ≠ *c*_*j*_} and *N*_*k*_1__(*i*) denotes the index set of the *k*_1_ nearest neighbours of sample *x*_*i*_ that are in the same class.

As can be seen from (1)-(2), the objective function of MFA is to look for an optimal transformation matrix *U* such that nearby data pairs in the same class are made close and the data pairs in different classes are separated from each other with the margin criterion. Therefore, maximizing it is an attempt to ensure both within-class compactness and between-class separability. Finally, the transformation matrices *U* of MFA are the eigenvectors associated with the largest eigenvalues of the following generalized eigenproblem:

(4) $$\begin{array}{c} X\left(D^{p}-W^{p}\right)X^{T}U=\lambda X\left(D-W\right)X^{T}U. \end{array}$$

*X*(*D* − *W*)*X* ^*T*^ is nonsingular after some preprocessing steps (such as PCA projection) on *X*; thus, the transformation matrix *U* of MFA can also be regarded as the eigenvectors of the matrix (*X*(*D*−*W*)*X* ^*T*^)^−1^ *X*(*D* ^*p*^ − *W* ^*p*^)*X* ^*T*^ associated with the largest eigenvalues.

Despite the success of applying MFA to many fields, there are still some problems that are not properly addressed till now.

1. MFA has a singular problem in face recognition, which stems from the fact that the number of training images is usually much smaller than the dimension of each image, a deficiency that is generally known as singular or small sample size (SSS) problem.
2. MFA is a supervised learning method; it needs a collection of labelled data in order to guarantee good generalization capability on testing samples. However, for real-world face recognition, it is easy to obtain a large number of face images while only a few of them are labelled manually. In this case, purely supervised MFA cannot be well trained because of the lack of sufficient labelled data.
3. MFA is still a linear technique in nature, so it is inadequate to describe the complexity of real face images because of illumination, facial expression, and pose variations. Although the nonlinear extension of MFA through kernel trick has been proposed in [19], the most commonly adopted kernels are the data-independent kernels which may not be consistent with the intrinsic manifold structure revealed by unlabeled data.

To fully address the above issues, we propose a novel semisupervised kernel MFA (SKMFA) algorithm for face recognition in the following section.

## 3. Semisupervised Kernel MFA Algorithm for Face Recognition

In the following, we first propose the semisupervised MFA algorithm which can avoid the singular problem and consider the unlabeled samples to learn the projection matrix for dimensionality reduction, and then the nonlinear extension of semisupervised MFA through kernel trick is proposed. Finally, we discuss how to design manifold adaptive nonparameter kernel function which can reflect the underlying geometry of the data.

### 3.1. Semisupervised MFA

Although MFA can produce linear discriminating features, the matrices *X*(*D* − *W*)*X* ^*T*^ and *X*(*D* ^*p*^ − *W* ^*p*^)*X* ^*T*^ in the generalized eigenproblem (4) are often singular because the number of available samples is smaller than the dimensionality of the samples. In order to avoid the numerical computational problem caused by matrix singularity, inspired by the scatter-difference-based discriminant analysis method [2, 31, 32], we modified the original objective function of MFA as

(5) J(U) =argmax(S~p−S~c) =argmax(∑i∑(i,j)∈Pk2(ci)  or  (i,j)∈Pk2(cj)||UTxi−UTxj||2Wijp−∑i∑i∈Nk1(j)  or  j∈Nk1(i)||WTxi−WTxj||2Wij) =argmaxTr(UTX(Dp−Wp)XTU−UTX(D−W)XTU) =argmaxTr(UT(X(Dp−Wp)XT−X(D−W)XT)U).

Maximizing *J*(*U*) is to find projections such that the close data points are attracted closer (minimizing the within-class compactness), while the data pairs in different classes are simultaneously separated from each other with the margin criterion (maximizing the between-class separability). So maximizing *J*(*U*) can be equivalently interpreted as minimizing the within-class compactness while simultaneously maximizing the between-class separability, which is consistent with the optimal objective of MFA.

In the formulation defined in (5), we have the freedom to multiply *U* by some nonzero constant. Thus, we additionally require *U* to be orthonormal vectors, which may help preserve the shape of the data distribution. This means that we need to solve the following constrained optimization problem:

(6) $$\begin{array}{c} \arg\underset{U}{\max}\;\mathrm{Tr}\left(U^{T}\left(X\left(D^{p}-W^{p}\right)X^{T}-X\left(D-W\right)X^{T}\right)U\right) \end{array}$$

(7) $$\begin{array}{c} \text{subject}\;\;\text{to}\;\;U^{T}U=I, \end{array}$$

where *I* is the identity matrix.

It is worth noting that the only differences between the previous optimization problem and the original optimization problem of MFA lie in the following: the former involves a constrained optimization whereas the latter solves an unconstrained optimization. The motivation for using the constraint *U* ^*T*^ *U* = *I* is that it allows us not to calculate the inverse of the matrix ${\tilde{S}}_{p}$ or ${\tilde{S}}_{c}$, which successfully avoids the matrix singularity problem existing in the original MFA.

In addition, the original MFA is a supervised learning technique, which typically requires a large number of training samples in order to achieve satisfactory performance. However, for the practical large-scale applications such as face recognition, one often faces a lack of sufficient labelled face image data since labelling often requires expensive human labour and much time. Meanwhile, large numbers of unlabeled face data can be far easier to obtain due to the rapid advances of digital camera technology. In this case, purely supervised dimensionality reduction algorithms cannot be well trained because of the lack of sufficient labelled data, and purely unsupervised methods are usually unreliable because there is no supervision guidance. This motivates us to explore semisupervised learning [20] techniques for dimensionality reduction. Consequently, to leverage both the labelled and unlabeled data for dimensionality reduction, we propose the semisupervised MFA algorithm as follows.

In face recognition, since the number of labelled samples is small, it is important to consider the unlabeled samples to learn the projection matrix for dimensionality reduction. In fact, recent research has found that unlabeled samples may be helpful to improve the classification performance [33, 34]. In the following, we generalize MFA by introducing new reconstruction optimizations based on unlabeled samples and then incorporating them into the whole dimensionality reduction process, which leads to the semisupervised MFA algorithm.

Assume that unlabeled samples are attached on the original data set: *X* = [*x*_1_, *x*_2_,…, *x*_*n*_, *x*_*n*+1_,…, *x*_*n*+*n*_*u*__], where the first *n* samples are labelled and the remaining *n*_*u*_ samples are unlabeled. For each unlabeled sample data *x*_*i*_  (*n* + 1 ≤ *i* ≤ *n* + *n*_*u*_), as in [12, 15, 33], we assume that all of its neighbourhoods are linear; that is, each data point can be optimally reconstructed using a linear combination of its neighbours. Hence, our objective is to minimize the reconstruction error:

(8) argminVij∑i=n+1n+nu||εi||2  =argminVij∑i=n+1n+nu||xi−∑n+1≤j≠i≤n+nuVijxj||2,

where *ε*_*i*_ is the reconstruction error for *x*_*i*_ and *V*_*ij*_ is the reconstruction coefficient which indicates the contribution of *x*_*j*_ to *x*_*i*_. We further constrain ∑_*n*+1≤*j*≠*i*≤*n*+*n*_*u*__ *V*_*ij*_ = 1, *V*_*ij*_ ≥ 0 on (8). Obviously, the more similar *x*_*j*_ is to *x*_*i*_, the larger *V*_*ij*_ will be. Hence, we can easily obtain *V*_*i*_ = ∑_*p*_ *C*_*i*,*p*_^−1^/∑_*p*,*q*_ *C*_*p*,*q*_^−1^, *C*_*p*,*q*_ = (*x*_*i*_−*x*_*p*_)^*T*^(*x*_*i*_ − *x*_*q*_) is the local Gram matrix, and *V*_*ij*_ is the *j*th elements of *V*_*i*_, wherein *n* + 1 ≤ *p*, *q*, *i* ≤ *n* + *n*_*u*_.

Then, we can reconstruct *y*_*i*_ ( = *U* ^*T*^ *x*_*i*_) from *y*_*j*_ ( = *U* ^*T*^ *x*_*j*_) in the low-dimensional feature space by using the obtained reconstruction coefficient *V*_*ij*_, wherein *n* + 1 ≤ *i*, *j* ≤ *n* + *n*_*u*_; that is,

(9) J(U)=argminU∑i=n+1n+nu||yi−∑n+1≤j≠i≤n+nuVijyj||2=argminU∑i=n+1n+nu||UTxi−∑n+1≤j≠i≤n+nuVijUTxj||2=argminU Tr(UTXu(I−VT)(I−VT)TXuTU),

where *Tr*() denotes the trace of matrix, *I* is the identity matrix, and *X*_*u*_ = [*x*_*n*+1_, *x*_*n*+2_,…, *x*_*n*+*n*_*u*__] is the data matrix to represent all unlabeled high-dimensional data samples.

Considering all the samples including both labelled and unlabeled samples, we obtain the whole optimal objective function of semisupervised MFA by using (6) and (9):

(10)  argmaxUTr(UT(X(Dp−Wp)XT−X(D−W)XT)U−τUTXu(I−VT)(I−VT)TXuTU)  =argmaxUTr   ×(UT((X(Dp−Wp)XT−X(D−W)XT)−τXu(I−VT)(I−VT)TXuT)U)  =argmaxUTr(UTMU)

(11) $$\begin{array}{c} \text{subject}\;\;\text{to}\;\;U^{T}U=I, \end{array}$$

where

(12) M=((X(Dp−Wp)XT−X(D−W)XT) −τXu(I−VT)(I−VT)TXuT)

and the parameter *τ* > 0 is a scaling factor to balance contributions to optimize *U* of labelled samples against unlabeled samples.

Obviously, the optimal projection matrices *U* of (10) are the eigenvectors associated with the largest eigenvalues of the following standard eigenproblem:

(13) $$\begin{array}{c} MU=\lambda U. \end{array}$$

Let the column vectors *U*_1_, *U*_2_,…, *U*_*d*_ be the solution of (13) ordered according to eigenvalues *λ*_1_ > *λ*_2_ > ⋯>*λ*_*d*_. The optimal projection matrix *U* is given by *U* = [*U*_1_, *U*_2_,…, *U*_*d*_]. Then, the embedding of the proposed semisupervised MFA is as follows:

(14) $$\begin{array}{c} x\to y=U^{T}x, \end{array}$$

where *y* is a lower-dimensional representation of the face image *x*.

Since the proposed semisupervised MFA does not need to compute any matrix inverse for generating discriminating lower-dimensional features, it successfully avoids the singularity problem existing in the original MFA.

### 3.2. Nonlinear Generalization of Semisupervised MFA via Kernel Trick

In this section, we describe how to generalize our proposed semisupervised MFA to the nonlinear case by using kernel trick [35]. The main idea of kernel trick is to map the input data to a feature space through a nonlinear mapping, where the inner products in the feature space can be computed by a kernel function without knowing the nonlinear mapping explicitly. Kernel trick has demonstrated huge success in modelling real-world data with highly complex nonlinear structures, such as support vector machine (SVM) [35], kernel linear discriminant analysis (KLDA) [36], and kernel principal component analysis (KPCA) [37].

To extend semisupervised MFA to the nonlinear case, which leads to semisupervised kernel MFA, we consider the problem in a feature space *F* induced by some nonlinear mapping

(15) $$\begin{array}{c} \varphi :{\mathbb{R}}^{n}\to F. \end{array}$$

For a proper chosen *φ*, an inner product 〈, 〉 can be defined in *F*, which makes for a so-called reproducing kernel Hilbert space (RKHS). More specifically,

(16) $$\begin{array}{c} K\left(x,y\right)=\langle \varphi \left(x\right),\varphi \left(y\right)\rangle . \end{array}$$

Holds, where *K*(, ) is a positive semidefinite kernel function. The popular kernel functions include Gaussian kernel, polynomial kernel, and Sigmoid kernel.

Let ${\tilde{S}}_{p}^{\varphi }$ and ${\tilde{S}}_{c}^{\varphi }$ denote the interclass separability and intraclass compactness in the feature space *F*, respectively. We have

(17) S~pφ=∑i∑(i,j)∈Pk2(ci) or (i,j)∈Pk2(cj)||UTφ(xi)−UTφ(xj)||2Wijp=Tr(UTφ(X)(Dp−Wp)φT(X)U)S~cφ=∑i∑i∈Nk1(j) or j∈Nk1(i)||WTxi−WTxj||2Wij=Tr(UTφ(X)(D−W)φT(X)U).

In addition, the reconstruction optimal function defined in (9) for unlabeled samples in the feature space can be transformed as follows:

(18) argminU∑i=n+1n+nu||yi−∑n+1≤j≠i≤n+nuVijyj||2 =argminU∑i=n+1n+nu||UTφ(xi)−∑n+1≤j≠i≤n+nuVijUTφ(xj)||2 =argminUTr(UTφ(Xu)(I−VT)(I−VT)TφT(Xu)U).

By combining (17) and (18) together, both labelled and unlabeled samples will be considered in obtaining the projection matrix *U* in the feature space. Then, we obtain the following optimal objective function of semisupervised kernel MFA:

(19) argmaxU(S~pφ−S~cφ−τ∑i=n+1n+nu||UTφ(xi)−∑n+1≤j≠i≤n+nuVijUTφ(xj)||2)=argmaxUTr(UTφ(X)(Dp−Wp)φT(X)U−UTφ(X)(D−W)φT(X)U−τUTφ(Xu)(I−VT)(I−VT)TφT(Xu)U)=argmaxUTr(UT(φ(X)(Dp−Wp)φT(X)−φ(X)(D−W)φT(X))U−τUTφ(Xu)(I−VT)(I−VT)TφT(Xu)U)=argmaxUTr(UTφ(X)((Dp−Wp)−(D−W))φT(X)U−τUTφ(Xu)(I−VT)(I−VT)TφT(Xu)U).

Because any solution *U* ∈ *F* must lie within the span of all the samples in *F*, there exist coefficients *α*_*i*_ (*i* = 1,2,…, *n*, *n* + 1,…, *n* + *n*_*u*_) such that

(20) $$\begin{array}{c} U=\overset{n+n_{u}}{\underset{i=1}{\sum }}\alpha_{i}\varphi \left(x_{i}\right)=\varphi \alpha , \end{array}$$

where *φ* = *φ*(*X*) = [*φ*(*x*_1_), *φ*(*x*_2_),…, *φ*(*x*_*n*+*n*_*u*__)] and *α* = (*α*_1_,*α*_2_,…,*α*_*n*+*n*_*u*__)^*T*^.

Following some algebraic formulations, we can rewrite (19) as follows:

(21) argmaxU(S~pφ−S~cφ−τ∑i=n+1n+nu||UTφ(xi)−∑n+1≤j≠i≤n+nuVijUTφ(xj)||2)  =argmaxUTr(UTφ(X)((Dp−Wp)−(D−W))φT(X)U−τUTφ(Xu)(I−VT)(I−VT)TφT(Xu)U)  =argmaxαTr(αTK((Dp−Wp)−(D−W))Kα−ταTKu(I−VT)(I−VT)TKuα)  =argmaxαTr(αT(K((Dp−Wp)−(D−W))K−τKu(I−VT)(I−VT)TKu)α),

where *K* is the kernel matrix defined on the labelled samples and *K*_*u*_ is the kernel matrix defined on the unlabeled samples.

By imposing *α* ^*T*^ *α* = *I* on (21), the problem of semisupervised kernel MFA (SKMFA) is transformed into finding the leading eigenvectors of matrix (*K*((*D* ^*p*^ − *W* ^*p*^) − (*D* − *W*))*K* − *τK*_*u*_(*I* − *V* ^*T*^)(*I*−*V* ^*T*^)^*T*^ *K*_*u*_). Since no matrix inverse needs to be computed, SKMFA successfully avoids the singularity problem.

Thus, each eigenvector *α* gives a projection function *U* in the feature space. For a new sample data *x*, its projection onto *U* in the feature space *F* can be calculated by

(22) 〈U,φ(x)〉=∑i=1nαi〈φ(xi),φ(x)〉=∑i=1n+nuαiK(xi,x),

where *K*(, ) is a given kernel function.

### 3.3. Manifold Adaptive Nonparameter Kernel

Similar to the other kernel-based methods, the kernel is also at the heart of semisupervised kernel MFA (SKMFA) algorithm. To achieve good performance, one has to define a good kernel presentation. However, the most commonly used kernels (such as Gaussian kernel, polynomial kernel, and Sigmoid kernel) are all data-independent kernels which may not be consistent with the intrinsic manifold structure revealed by unlabeled data points [38]. Meanwhile, these traditional kernels need complex operation to determine model parameters, which greatly limits their performance. To tackle the previous problems, a novel manifold adaptive nonparameter kernel function is proposed to improve the performance of SKMFA.

Let *v* be a linear space with a positive semidefinite inner product (quadratic from) and let *S* : *H* → *v* be a bounded linear operator. We define $\tilde{H}$ to be the space of functions from *H* with the modified inner product

(23) $$\begin{array}{c} {\langle f,g\rangle }_{\tilde{H}}={\langle f,g\rangle }_{H}+{\langle Sf,Sg\rangle }_{v}. \end{array}$$

It has been shown that $\tilde{H}$ is still a reproducing kernel hilbert space (RKHS) [38].

Given the data points *x*_1_, *x*_2_,…, *x*_*n*_, let *S* : *H* → ℝ^*n*^ be the evaluation map *S*(*f*) = (*f*(*x*_1_), *f*(*x*_2_),…, *f*(*x*_*n*_)). Denote *f* = (*f*(*x*_1_), *f*(*x*_2_),…, *f*(*x*_*n*_)), *g* = (*g*(*x*_1_), *g*(*x*_2_),…, *g*(*x*_*n*_)). Note that *f*, *g* ∈ *v*; thus we can obtain

(24) $$\begin{array}{c} {\langle Sf,Sg\rangle }_{v}=\langle f,g\rangle =f^{T}Mg, \end{array}$$

where *M* is a symmetric positive semi-definite matrix. If we define

(25) $$\begin{array}{c} K_{x}=\left(K\left(x,x_{1}\right),\ldots ,K\left(x,x_{n}\right)\right), \end{array}$$

then it can be shown that the reproducing kernel $\tilde{K}$ in $\tilde{H}$ is of the following explicit form:

(26) $$\begin{array}{c} \tilde{K}\left(x,z\right)=K\left(x,z\right)-K_{x}^{T}{\left(I+MK\right)}^{-1}MK_{z}, \end{array}$$

where *I* is an identity matrix and *K* is the kernel matrix *K*_*ij*_ = *K*(*x*_*i*_, *x*_*j*_) in *H*. The key issue now is the choice of *M* and the original kernel *K*, so that the deformation of kernel induced by the data-dependent norm is motivated with respect to the intrinsic geometry of the data.

In order to model the intrinsic manifold structure, as suggested in [16], the graph Laplacian implements a smoothness assumption with respect to an empirical estimate of the geometric structure of the data. Then we construct a nearest graph *G*(*V*, *W*) to reflect the underlying manifold structure of the data. Each data point *x*_*i*_ corresponds to a node in *V*, and an edge is established between two nodes *v*_*i*_ and *v*_*j*_ if the corresponding two data points *x*_*i*_ and *x*_*j*_ are among *k* nearest neighbours of each other. Although there are many choices for the weight matrix on the graph, in order to eliminate the noise data on the manifold, we adopt the trick proposed in [39] to construct the nearest graph *G*. Let us define a distance function *h*(*x*) = ||*x* − *x* ^(*k*)^||, where *x* ^(*k*)^ is the *k*th nearest neighbor of *x* in *G*. The weight matrix *W* associated with *G* is defined as follows:

(27) $$\begin{array}{c} W_{ij}=\{\begin{array}{cc} \exp\left(-\frac{{||x_{i}-x_{j}||}^{2}}{\max\left\{h^{2}\left(x_{i}\right),h^{2}\left(x_{j}\right)\right\}}\right), & \\ \;\text{if}\;||x_{i}-x_{j}||\leq \max\left\{h\left(x_{i}\right),h\left(x_{j}\right)\right\} & \\ 0, & \\ \;\text{otherwise}. & \end{array} \end{array}$$

The graph Laplacian is defined as *L* = *D* − *W*, where *D* is a diagonal degree matrix given by *D*_*ii*_ = ∑_*j*_ *W*_*ij*_. The graph Laplacian provides the following smoothness penalty on the graph:

(28) $$\begin{array}{c} f^{T}Lf=\overset{n}{\underset{i=1}{\sum }}\left(f\left(x_{i}\right)-f\left(x_{j}\right)\right)W_{ij}. \end{array}$$

Thus, we set *M* in (26) to be *M* = *L*. Then, the next central issue is how to select an original input kernel function. The traditionally used kernel functions often assume certain parametric forms, but how to choose appropriate parameters of kernel function is an open problem, thus limiting their capacity of fitting diverse patterns in real applications. To cope with that problem, we adopt the following nonparameter kernel function as the original input kernel function in (26).

Generally, a nonparameter kernel matrix *K* with respect to *n* patterns can be expressed as *K* = *X* ^*T*^ *X*≻0, where *X* = [*x*_1_, *x*_2_,…, *x*_*n*_] is the matrix of the embedding of data points [40]. The regularizer of the kernel matrix *K*, which captures the local dependency between the embedding of *x*_*i*_ and *x*_*j*_, can be defined as

(29) ∑i,j=1nWij||xiDii−xjDjj||2  =Tr(XL~XT)=Tr(L~K),

where $\tilde{L}$ is the normalized graph Laplacian matrix defined as follows:

(30) $$\begin{array}{c} \tilde{L}=I-D^{-1/2}WD^{-1/2}, \end{array}$$

where *W* is defined in (27) and *D* is a diagonal degree matrix given by *D*_*ii*_ = ∑_*j*_ *W*_*ij*_.

In addition, for the semisupervised learning algorithm, recent researches have pointed out that the class label information is not readily available, while it is easier to obtain some collection of similar pairwise constraints *S* (known as “must links,” i.e., the data pairs share the same class label) and a collection of dissimilar pairwise DS (known as “cannot-links,” i.e., the data pairs have different class label), which is often referred to as “side information.” Given *S* and DS, we construct a similarity matrix *T* ∈ ℝ^*n*×*n*^ to represent the pairwise constraints; that is,

(31) $$\begin{array}{c} T_{ij}=\{\begin{array}{cc} +1, & \left(x_{i},x_{j}\right)\in S\\ -1, & \left(x_{i},x_{j}\right)\in \text{DS}\\ 0, & \text{otherwise.} \end{array} \end{array}$$

Then, an intuitive principle for kernel learning is that the kernel entry *K*_*ij*_ = *K*(*x*_*i*_, *x*_*j*_) should be aligned with the side information *T*_*ij*_ as much as possible; that is, the alignment *T*_*ij*_ *K*_*ij*_ of each kernel entry is maximized.

Therefore, following the suggestions in [40], by simultaneously considering the side information in (31) and the regularizer in (29), the nonparameter kernel learning can be formulated as follows:

(32) $$\begin{array}{c} \underset{K≻0}{\min}\mathrm{Tr}\left(\tilde{L}K\right)+C\sum_{(x_{i},x_{j})\in S\cup \text{DS}}l\left(T_{ij}K_{ij}\right), \end{array}$$

where *C* is the positive constant to control the tradeoff between the empirical loss *l*() and the intrinsic data manifold and *l*() is the square hinge loss function defined as follows:

(33) $$\begin{array}{c} l\left(f\right)=\frac{1}{2}{\left(\max\left(0,1-f\right)\right)}^{2}. \end{array}$$

It is worth noting that the previous optimization problem belongs to a semidefinite programming (SDP) problem, which can be solved with standard SDP solver SeDuMi [41]. Once we obtain the optimal nonparameter kernel *K*, by substituting *M*  ( = *L*) and *K* into (26), we eventually get the following manifold kernel function:

(34) $$\begin{array}{c} \tilde{K}\left(x,z\right)=K\left(x,z\right)-K_{x}^{T}{\left(I+LK\right)}^{-1}LK_{z}. \end{array}$$

### 3.4. The Semisupervised Kernel MFA Algorithm

We summarize our proposed semisupervised kernel MFA (SKMFA) algorithm as follows.

(1) Calculate the initial nonparameter kernel matrix *K* by solving the optimization problem (32).
(2) Compute the weight matrix *W* in terms of (27) and set *M* = *L*, where *L* = *D* − *W* is graph Laplacian and *D* is a diagonal degree matrix given by *D*_*ii*_ = ∑_*j*_ *W*_*ij*_.
(3) Obtain the manifold adaptive kernel $\tilde{K}$ according to (34).
(4) Find the eigenvector *α* by solving the following eigenproblem:
  (35) $$\begin{array}{c} \left(\begin{array}{c} \tilde{K}\left(\left(D^{p}-W^{p}\right)-\left(D-W\right)\right)\tilde{K}\\ -\tau {\tilde{K}}_{u}\left(I-V^{T}\right){\left(I-V^{T}\right)}^{T}{\tilde{K}}_{u} \end{array}\right)\alpha =\lambda \alpha . \end{array}$$
(5) For a new sample data *x*, its lower-dimensionality representation *y* can be calculated by
  (36) $$\begin{array}{c} x\to y=\overset{n+n_{u}}{\underset{i=1}{\sum }}\alpha_{i}\tilde{K}\left(x_{i},x\right), \end{array}$$
  where $\tilde{K}\left(,\right)$ is the manifold adaptive kernel defined in (34).

## 4. Experimental Results

In this section, we investigate the performance of our proposed semisupervised kernel MFA (SKMFA) algorithm and compare it with other representative dimensionality reduction algorithms for face recognition. All of our experiments have been performed on a P4 3.5 GHz Windows XP machine with 2 GB memory.

### 4.1. Face Databases and Experimental Settings

Three real-world face databases are used in our experimental study, including the Yale database, the Olivetti Research Laboratory (ORL) database, and the PIE (pose, illumination, and expression) database from CMU. In all experiments, preprocessing to locate the faces was applied. Original face images were manually aligned, cropped, and then resized to 32 × 32 pixels, with 256 gray levels per pixel. The important statistics of three databases are summarized next.

**Figure 1:**
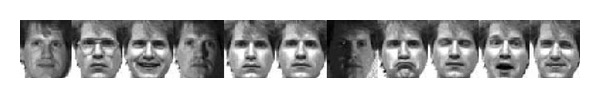
Face image examples of the Yale database.

The Yale face database (http://cvc.yale.edu/projects/yalefaces/yalefaces.html) was constructed at the Yale centre for computational vision and control. There are 15 persons, and each person has 11 different images. The images demonstrate variations in lighting condition and facial expression. Some sample face images after preprocessing of the database are shown in Figure 1.

**Figure 2:**
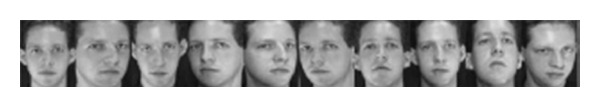
Face image examples of the ORL database.

In the ORL database (http://www.cl.cam.ac.uk/research/dtg/attarchive/facedatabase.html), there are 40 persons, and each person has 10 different images. Some images were captured at different times and have different variations including expression and facial details. Some sample face images after preprocessing of the database are shown in Figure 2.

**Figure 3:**
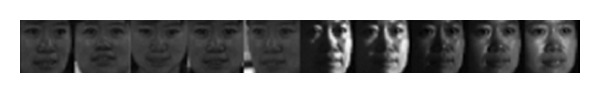
Face image examples of the CMU PIE database.

The CMU PIE database [42] contains 68 individuals with 41368 face images as a whole. The face images were captured by 13 synchronized cameras and 21 flashes under varying poses, illumination, and expressions. In this experiment, we choose the five frontal poses (C05, C07, C09, C27, and C29) and illumination indexed as 10 and 13 which contain 10 front face images for each person. Some sample face images after preprocessing of the database are shown in Figure 3.

The experimental settings are as follows. We randomly selected 10 face images per individual to form the training set for Yale database and 8 face images per individual to form the training set for ORL and CMU PIE databases. The remaining images for each person were used for the testing set. In the training set, we randomly selected *l*  ( = 2,4) face images per individual as labelled data set and the rest as unlabeled data set for each face image database.

To perform face recognition, we first obtain the face subspace by using dimensionality reduction algorithms. Then, the new face images to be identified are projected onto the face subspaces. Finally, the nearest-neighbour classifier is adopted to identify new facial images, where the Euclidean metric is used as the distance measure.

### 4.2. Compared Algorithms

Five algorithms, which are compared in our experiments, are listed below:

1. Marginal Fisher analysis (MFA) [19], which provides us with a baseline performance of linear dimensionality reduction algorithms. We can examine the usefulness of kernel approaches by comparing the performance of kernel Marginal Fisher analysis with MFA.
2. Kernel marginal Fisher analysis (KMFA) is proposed in [19]. This method is the nonlinear extension of the traditional MFA via kernel trick. The settings of KMFA algorithm are identical to the description in the corresponding paper [19].
3. Semisupervised discriminant analysis (SDA) is proposed in [30], which is believed to be one of the most representative semisupervised dimensionality reduction algorithms.
4. Kernel semisupervised discriminant analysis (KSDA) is proposed in [30]. This method is the nonlinear extension of the traditional SDA via kernel trick. The settings of KSDA algorithm are identical to the description in the corresponding paper [30].
5. Semisupervised kernel marginal Fisher analysis (SKMFA), as described in Section 3, is a new method proposed in this paper.

Note that the settings of the compared algorithms are identical to the description in the corresponding papers. For our proposed SKMFA algorithm, there is a parameter *τ* which controls the balance contributions of labelled samples against unlabeled samples. We simply set the value of *τ* as 1, and the effect of parameter selection will be discussed later.

### 4.3. Face Recognition Results

**Figure 4:**
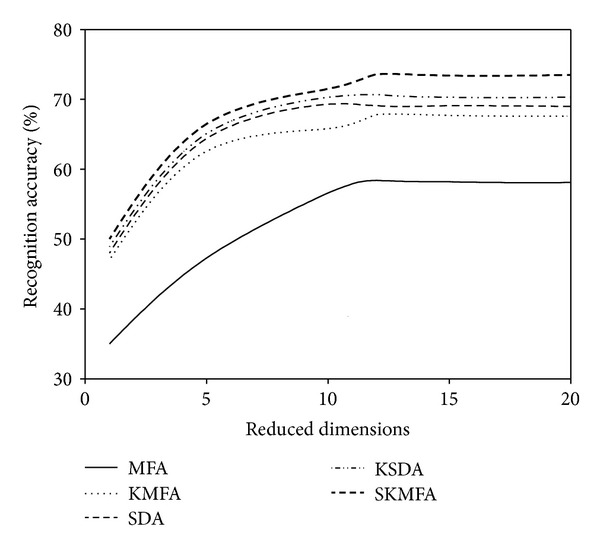
Two labelled data for training on the Yale database.

**Figure 5:**
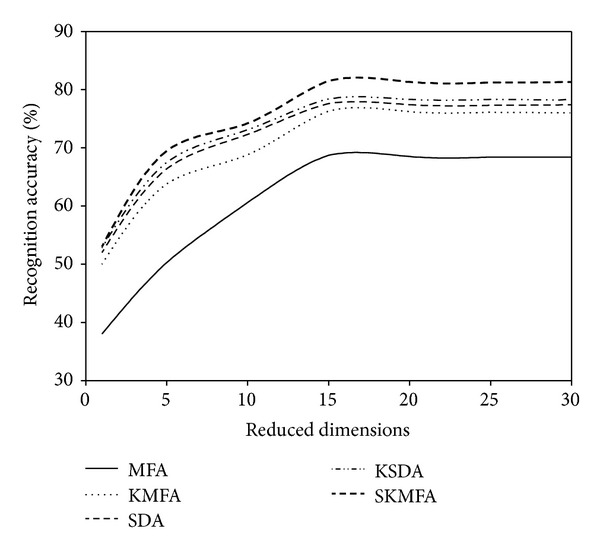
Four labelled data for training on the Yale database.

**Figure 6:**
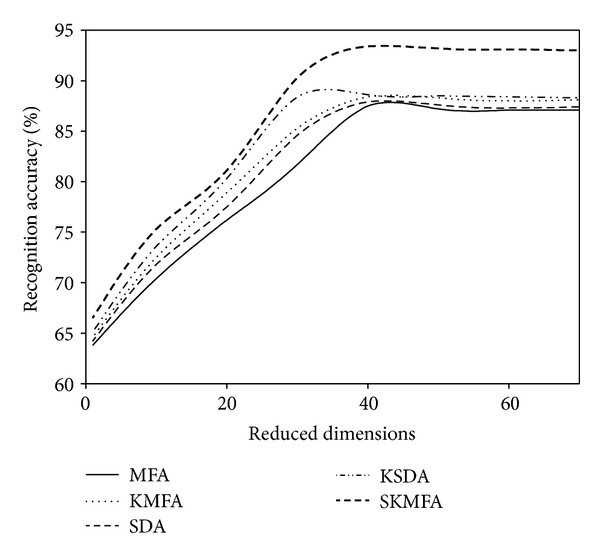
Two labelled data for training on the ORL database.

**Figure 7:**
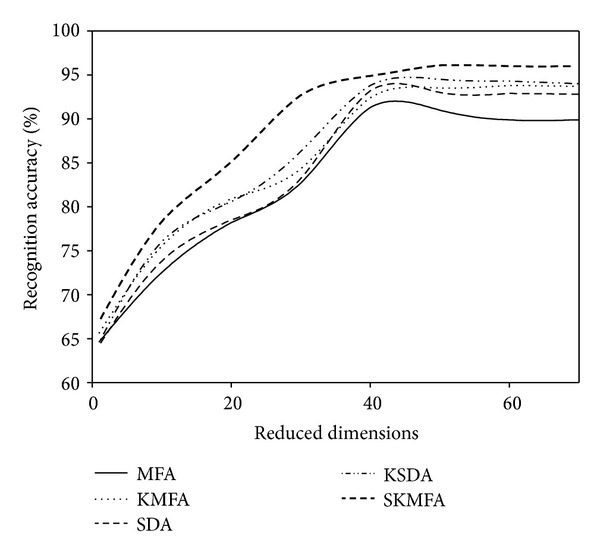
Four labelled data for training on the ORL database.

**Figure 8:**
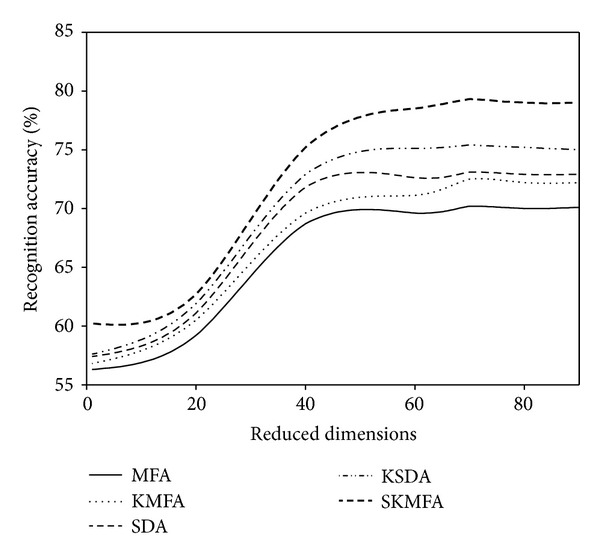
Two labelled data for training on the CMU PIE database.

**Figure 9:**
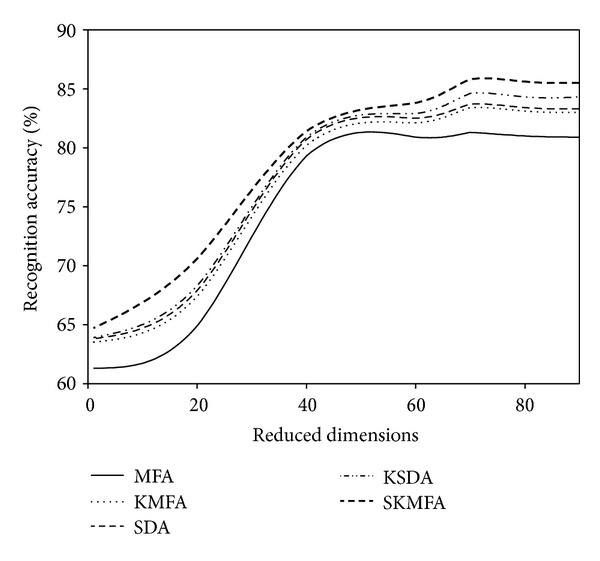
Four labelled data for training on the CMU PIE database.

For each given *l*  ( = 2,4), we average the results over 20 random splits and report the mean as well as the standard deviation. The face recognition accuracies for each algorithm in three face databases are reported on the Tables 1, 2, and 3, respectively. The recognition accuracies versus the variation of reduced dimensions are shown in Figures 4, 5, 6, 7, 8, and 9. The main observations from the previous performance comparisons include the following.

1. Our proposed SKMFA algorithm consistently outperforms the MFA, KMFA, SDA, and KSDA algorithms in terms of recognition accuracy, which indicates that SKMFA can effectively use the intrinsic nonlinear manifold structure revealed by unlabeled data to improve the recognition accuracy.
2. The KMFA and KSDA algorithms achieve higher recognition accuracy than their linear counterparts (i.e., MFA and SDA), respectively, which suggests the effectiveness of kernel approaches.
3. The semisupervised algorithms (SKMFA, KSDA, and SDA) achieve higher recognition accuracy than the supervised algorithm (MFA), which demonstrates that these semisupervised algorithms can effectively utilize only a few labelled samples to predict the labels of the unlabeled samples.
4. The recognition accuracies of SDA and KMFA are almost similar. For some databases, SDA outperforms KMFA, while KMFA is better than SDA for other databases. A possible explanation is as follows: KMFA is only a supervised nonlinear algorithm (not a semisupervised algorithm), while SDA is only a semisupervised linear algorithm (not a nonlinear algorithm). Thus, it is hard to evaluate whether nonlinear extension with kernel trick or semisupervised information is more important for recognition.
5. Although KSDA and KMFA belong to kernel-based nonlinear manifold learning algorithm, KSDA performs better than KMFA. The possible explanation is that KSDA can utilize a large number of unlabeled data as well as relatively limited labelled data for better discrimination ability.
6. Although KMFA and SKMFA are the nonlinear extensions of MFA via kernel trick, KMFA performs much worse than SKMFA. This is because KMFA adopts the commonly used data-independent kernel which may not be consistent with the intrinsic manifold structure of face images.
7. Our proposed SKMFA algorithm achieves much better performance than the MFA, KMFA, SDA, and KSDA algorithms. The main reason could be attributed to the following fact: first, SKMFA simultaneously considers the intraclass geometry and the interactions of samples from different classes; second, SKMFA successfully avoids the singularity problem without calculating the matrix inverse; third, the manifold adaptive kernel is consistent with the intrinsic manifold structure revealed by unlabeled data points and can effectively capture the nonlinear structure of face images. Therefore, our proposed SKMFA algorithm achieves the best performance among the compared algorithms by simultaneously using the aforementioned optimal strategies.

### 4.4. Parameter Selection for SKMFA

**Figure 10:**
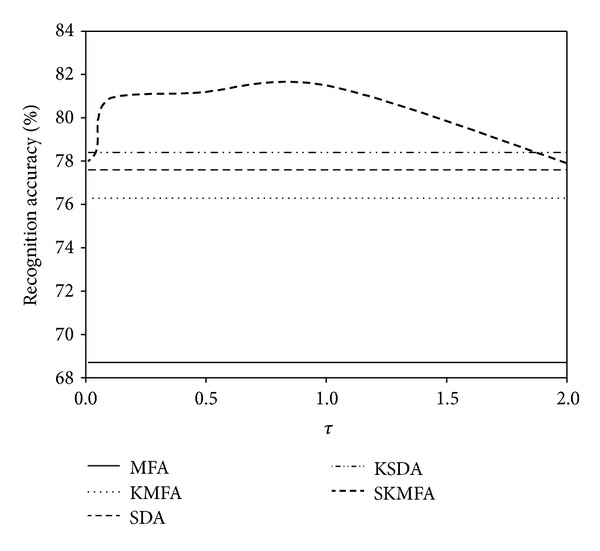
The performance of SKMFA varies with the parameter *τ* on the Yale database.

**Figure 11:**
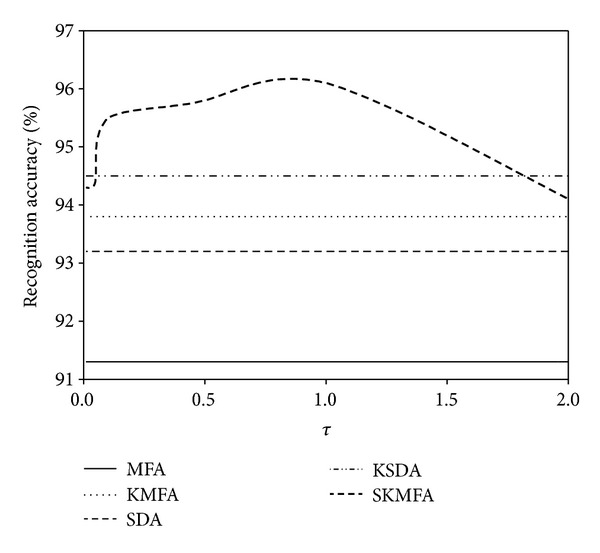
The performance of SKMFA varies with the parameter *τ* on the ORL database.

**Figure 12:**
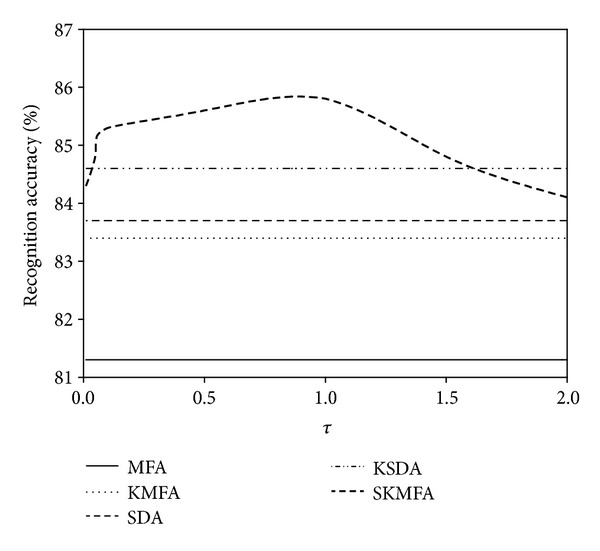
The performance of SKMFA varies with the parameter *τ* on the CMU PIE database.

The parameter *τ* > 0 is an essential parameter in our SKMFA algorithm which controls the balance contributions of labelled samples against unlabeled samples. We empirically set it to be 1 in the previous experiments. In this subsection, we try to examine the impact of parameter *τ* on the performance of SKMFA. Figures 10, 11, and 12 show how the average performance of SKMFA varies with *τ*.

## 5. Conclusions

In this paper, we have proposed a novel nonlinear algorithm, called semisupervised kernel marginal Fisher analysis (SKMFA), for face recognition. It can make efficient use of both labelled and unlabeled data points for nonlinear dimensionality reduction. The labelled data points are used to maximize the discriminating power, while the unlabeled data points are used to reveal the intrinsic manifold structure. In addition, the manifold adaptive kernel is adopted to further improve the algorithm performance. Experimental results on three face image databases demonstrate the effectiveness of our proposed algorithm. Since our proposed SKMFA algorithm is a general nonlinear dimensionality reduction algorithm for high-dimensional data, we plan to apply the algorithm to video and audio classification in the future.

## Figures and Tables

**Table 1:** Comparisons of recognition accuracy (in percent) on the Yale database.

Algorithm	Two labels	Four labels
MFA	58.4 ± 1.15	68.7 ± 1.27
KMFA	67.8 ± 1.28	76.3 ± 1.33
SDA	69.3 ± 1.49	77.6 ± 1.25
KSDA	70.7 ± 1.32	78.4 ± 1.18
SKMFA	73.6 ± 0.73	81.5 ± 0.69

**Table 2:** Comparisons of recognition accuracy (in percent) on the ORL database.

Algorithm	Two labels	Four labels
MFA	87.5 ± 1.43	91.3 ± 1.41
KMFA	88.4 ± 1.50	93.8 ± 1.38
SDA	87.9 ± 1.56	93.2 ± 1.42
KSDA	88.6 ± 1.37	94.5 ± 1.29
SKMFA	93.4 ± 1.25	96.1 ± 1.07

**Table 3:** Comparisons of recognition accuracy (in percent) on the CMU PIE database.

Algorithm	Two labels	Four labels
MFA	70.2 ± 1.90	81.3 ± 1.76
KMFA	72.5 ± 1.85	83.4 ± 1.72
SDA	73.1 ± 1.63	83.7 ± 1.49
KSDA	75.4 ± 1.27	84.6 ± 0.98
SKMFA	79.3 ± 0.98	85.8 ± 0.75
